# Sleep-wake cycle on amplitude-integrated EEG and neuroimage outcomes in newborns

**DOI:** 10.1186/s13052-016-0294-z

**Published:** 2016-09-15

**Authors:** Seonkyeong Rhie, Kyu Young Chae, Heui Seung Jo, Kyu Hyung Lee

**Affiliations:** Department of Pediatrics, CHA Bundang Medical Center, CHA University, 59 Yatap-ro, Bundang-gu, Seongnam-si, Gyeonggi-do 463-712 South Korea

**Keywords:** Infant, Cyclicity, Sleep-wake cycle, aEEG, Amplitude-integrated EEG

## Abstract

**Background:**

The aim of this study was to evaluate the results of sleep-wake cycle monitoring using amplitude-integrated EEG (aEEG) and neuroimaging in newborn infants with a possible perinatal hypoxic insult, investigate the correlation between the findings, and determine the relevance of the findings to reasonably predict neurological outcome.

**Methods:**

aEEG was recorded among newborn infants suspected of perinatal asphyxia between November, 2014 and June, 2015 in one neonatal intensive care unit facility. Brain imaging with serial ultrasonography and MRI when available were performed, and the infants were divided into two groups according to findings and potential neurological outcome: Group I (favorable findings) and Group II (severe findings such as high grade intraventricular hemorrhage, cerebral infarction or white matter injury). Established sleep-wake cycle times after birth was compared between the two groups.

**Results:**

Among 107 newborn infants, 85 subjects were classified as Group I and the remaining 22 subjects as Group II. The total number of aEEG sessions was 207 and recording time was 2,796 h with a mean of 14.43 ± 13.40 h per study. Estimated times of cyclicity were earlier in Group I (113.34 h, 95 % CI 82.31–144.37) as compared to Group II (504.39 h, 95 % CI 319.91–688.88; *p* < 0.001).

**Conclusions:**

Delayed cyclicity on aEEG has a strong correlation with unfavorable brain neuroimages in newborns with possible perinatal asphyxia. If sleep-wake cycles do not appear during initial period after birth, follow-up aEEG studies are recommended.

**Trial registration:**

Retrospectively registered

Registration number: BD 2015–148

Name of registry: amplitude integrated EEG in neonate

Date of registration: September 9, 2015

## Background

Amplitude-integrated EEG (aEEG) is a simple, real-time method for monitoring newborn brain function [1, 2], which generates a considerable amount of information about an infant’s neurological status and prognosis by classifying background activity and assessing the presence of seizures [3–5]. In severely asphyxiated neonates, all persistent and most initial flat traces or burst suppression in background activity on aEEG correlates with severe deficits at 24 months of age [4], and the presence of seizure activity after birth is associated with poor neurodevelopmental outcome [6]. Therefore, identification of normal versus abnormal developmental electrocortical findings on aEEG may reveal the neurological status of newborns, as well as a time window for neuroprotective intervention. The onset of the electrical sleep-wake cycle on aEEG has been reported to be an important determining prognostic factor in neurological at-risk neonates [5, 7]. However, only few studies have evaluated the longitudinal course of aEEG tracings to determine the onset of cyclicity.

In this study, we used aEEG to evaluate the relationship between establishment of sleep-wake cycle and neuroimage findings in newborn infants with suspected perinatal asphyxia treated in the neonatal intensive care unit (NICU). Specifically, we sought to 1) determine whether establishing a sleep-wake cycle, as indicated by in electrocortical activity patterns, can predict normal brain development, and 2) identify the optimal time window to perform follow-up studies and determine a prognosis after an initial aEEG recording without cyclicity.

## Methods

### Subjects

**Fig. 1:**
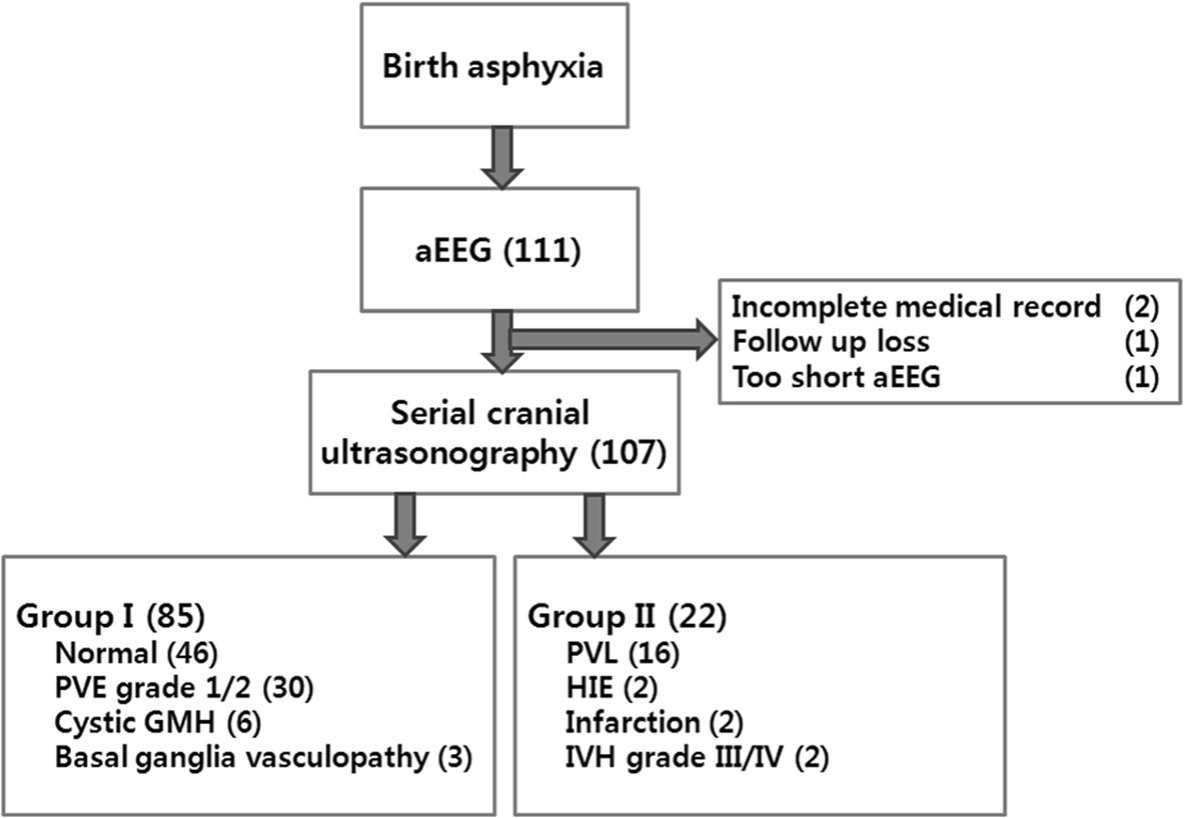
Flow diagram shows patient categorization. Among 111 patients, serial cranial ultrasonographies were performed in 107. In Group I, basal ganglia vasculopathy, cystic GMH, PVE grade 1/2, and normal findings were included. Group II were composed with severe findings included PVL, IVH grade III/IV, HIE and infarction. aEEG, amplitude-integrated electroencephalogram; GMH, germinal matrix haemorrhage; HIE, hypoxic ischemic encephalopathy; IVH, intraventricular haemorrhage; PVE, periventricular echodensity; PVL, periventricular leukomalacia

The study was conducted in the NICU of CHA Bundang Medical Center between November, 2014 and June, 2015. We enrolled inborn and outborn newborns with a gestational age (GA) of over 26 weeks. We performed aEEG recordings in newborns with suspected perinatal asphyxia with 5 min Apgar score <7 who needed positive pressure ventilation at delivery room, and also in infants with 5 min Apgar score ≥7 with decreased activity immediately after birth and presented with clinical seizure before 6 h after birth. Exclusion criteria were definite chromosomal anomalies, severe brain trauma after establishing cyclicity, urgent management other than aEEG monitoring, and metabolic disorders. GA was determined by obstetrical estimates based on the last menstrual cycle and ultrasonographic measurement during the first trimester. The 107 participating patients were divided into two groups based on neuroimaging findings. If there were follow up imaging studies, the most recent was used. Group I was infants with favorable brain image findings related to good neurologic outcomes [8–10] which included normal findings, increased periventricular echodensity grade 1 or 2 [11], small cystic germinal matrix hemorrhage and suspected basal ganglia vasculopathy without definite change (Fig. 1). Group II was infants with severe brain image findings related with poor neurological outcomes including: periventricular leukomalacia, intraventricular hemorrhage grade III or IV [12], ventriculomegaly to hydrocephalus, hypoxic ischemic encephalopathy, and cerebral infarction (Fig. 1).

### Data acquisition

Two- or four-channel aEEG monitoring was recorded from biparietal (P3 and P4) areas and ground (Fz) with or without bifrontal (F3 and F4) electrodes. Monitoring started within the first 48 h after birth or admission. If aEEG recording did not show a mature sleep-wake cycle (cyclicity), additional recordings were taken 48 to 72 h after the first recording and every 1 or 2 weeks thereafter until cyclicity developed. Monitoring was done with either the Electromed Cerebral Function Monitor System 5330 CFM (Olympic Medical, Seattle, WA, USA) or the EEG-1250 (Nihon Kohden, Tokyo, Japan). The specific device used for each patient was enrolled randomly.

Initial cranial ultrasonography was performed on all patients within 5 days after birth and results confirmed by a pediatric radiology specialist. Follow up studies were performed every 2–3 weeks until 2–4 months after birth. Brain MRI was additionally performed to selective cases before discharge if it was available and judged to be effective to determine an infant’s neurologic status. Patients were grouped based on the last ultrasonography or brain MRI.

### Data analysis

All aEEG data were divided according to the time after birth when the recordings were obtained: 0–6 h, 6–12 h, 12–24 h, 24–48 h, 48–72 h, 72–144 h, 7^th^–10^th^ day, 11^th^–14^th^ day, and all remaining time intervals. Birth times were based on medical records, except for outborn patients for whom birth times were estimated by initial physical exam, initial urination, or initial vitamin K injection time. Recordings shorter than 60 min were discarded because the session was too short to identify cyclicity. All sessions were individually scored. The presence of sleep-wake cyclicity was classified as: no cycling (score 0), waves first appear (score 1), not definite (score 2), definite cycling with (score 3) and without (score 4) interruption, and mature cycling (score 5) [13]. Recordings with a cyclicity score ≥3 (definite cycling with or without interruption and mature cycling) were considered to contain a sleep-wake cycle. The time taken to establish sleep-wake cyclicity after birth was defined as the interval from birth time to the initial appearance of a sleep wake cycle score over 3.

### Statistical analysis

Standard statistical methods were used. Survival curves were estimated with the Kaplan-Meier product-limit method and were compared using log-rank tests. The Cox proportional hazard model was used to define differences in cumulative probability between groups with SPSS (version 21.0, IBM) and R statistical software (version 3.2.2). Patients were censored at the last aEEG follow up.

## Results

A total of 107 patients participated in this single-center study. Initially, we performed aEEGs on 111 newborn infants with suspected neurological complications based on evidence of insults such as birth asphyxia or hypoxemia in their history or upon physical examination. Among them, 4 newborns were excluded due to incomplete medical records (*n* = 2), loss to follow up cranial ultrasonography (*n* = 1), or incomplete aEEG recordings (*n* = 1) (Fig. 1). In the 107 remaining patients, a total of 207 aEEG sessions were recorded, however 3 sessions were excluded due to poor quality. The total recording time was 2796 h with a mean of 14.43 ± 13.40 h per study.

The characteristics of the 107 subjects are summarized in Table 1. Overall, 85 subjects were defined as Group I. The remaining 22 subjects were Group II (Fig. 1). Brain MRI was studied in only one and six patients in Group I and Group II, respectively. We found that 73 patients in Group I (85.9 %) and 14 patients in Group II (63.6 %) exhibited cyclicity; the corresponding numbers of censored patients per each group were 12 and 8, respectively. The male to female ratios were not different between the two groups (Table 1). The 1- and 5-min Apgar scores in Group I were significantly higher than those in Group II. (*p* < 0.001 and *p* = 0.03 respectively) (Table 1). Group II was comprised of more premature subjects than Group I (34.0 ± 4.4 weeks and 37.2 ± 2.3 weeks, *p* = 0.003).

**Table 1:** Baseline characteristics stratified according to neuroimaging outcome

		Group I^a^ (*N* = 85)	Group II^b^ (*N* = 22)	*p*-value
Sex				
Male		40 (47 %)	13 (59 %)	0.350
Gestational age (week)		37.2 ± 2.3	34.0 ± 4.4	0.003
< 32 weeks (n)		1	8	
32–36 weeks (n)		38	8	
≥ 37 weeks (n)		46	6	
Body weight at birth (g)		2700 ± 680	2090 ± 840	<0.001
Apgar score	1 min	8 (7–8)	6 (5–7)	<0.001
	5 min	9 (8–9)	8 (7–8)	0.001

Data are n (%), mean ± standard deviation, or median (IQR)

^a^infants with favorable brain image finding

^b^infants with severe brain image finding

**Fig. 2:**
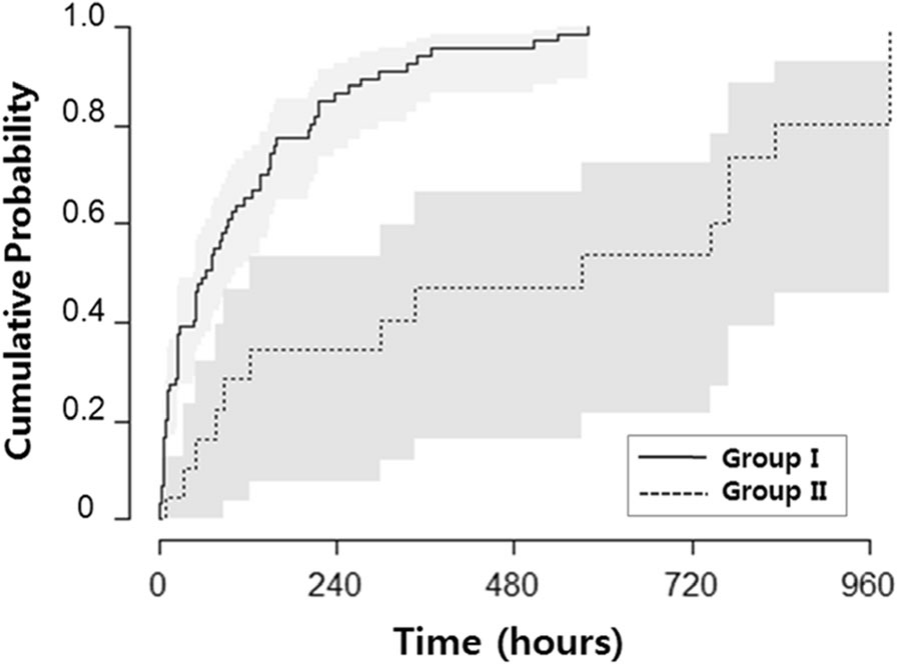
Estimated cumulative probability of cyclicity between Groups I and II (95 % confidence intervals (CI) are presenting as *gray zone*). Estimated time to sleep-wake cyclicity according to Groups I and II were derived from Cox regression models. Group I was infants with favorable brain image findings and Group II was infants with severe brain image findings. The *solid line* is Group I and *gray zone* indicates 95 % CI. The *dot line* indicates Group II. Cumulative probability of cyclicity is higher in Group I compared to Group II. Between the two groups, differences are significant after about 240 h after birth

In Group I, cyclicity was detected earlier than in Group II (*p* < 0.001). The time to cyclicity in Group I was 94 ± 125 h, estimated to be 113.34 (95 % confidence interval (CI) 82.31–144.37) hours. The corresponding values for Group II were 356 ± 365 h, estimated to be 504.39 (95 % CI 319.91–688.88) hours (*p* = 0.003 and *p* < 0.001, respectively). In Group I, 25 ± 5 %, 38 ± 5 %, 44 ± 6 %, 54 ± 6 %, 70 ± 5 %, 77 ± 5 %, and 86 ± 4 % of subjects were estimated to show cyclicity within 12, 24, 48, 72, 144, 168, and 240 h of birth, respectively (Fig. 2). Conversely, in the Group II, just 17 ± 9 % and 34 ± 12 % exhibited cyclicity within 72 and 144 h after birth, respectively. Therefore, differences in the estimated probability between the two groups were the highest 5 days after the infant’s birth, when the rate of cyclicity in Group II was only 30 % of the rate Group I (*p* = 0.002). The proportion of cyclicity increased by 13 % for every week of GA (*p* = 0.01) (Table 2). However, the rates of cyclicity were not related to sex (*p* = 0.43), initial cranial ultrasonographic findings (*p* = 0.80), or 1-min (*p* = 0.36) or 5-min (*p* = 0.34) Apgar scores.

**Table 2:** Prognostic effect attributable to cyclicity

	Hazard ratio	(95 % CI)	*p*-value
Female^a^	1.214	(0.750–1.964)	0.430
Group I (favorable image)^b^	3.378	(1.563–7.299)	0.002
Gestational age (week)	1.130	(1.028–1.243)	0.012
1 min AS ≥7^c^	0.847	(0.593–1.209)	0.360
5 min AS ≥7^c^	1.193	(0.828–1.720)	0.343

*CI* confidence interval, *AS* Apgar score

Compared with reference: ^a^male, ^b^group II (severe brain image), ^c^Apgar score ≤ 6

## Discussion

This study demonstrates that earlier sleep-wake cyclicity was found in the favorable neuroimaging group as compared to the severe neuroimaging group among the newborns with suspected perinatal asphyxia. Among various wave patterns of aEEG, sleep-wake cycle status has been suggested as an accurate predictor for the neurologic outcome in previous studies [14, 15]. A mature sleep-wake pattern presents in preterm infants older than 28 weeks of gestation [16]. On the other hand, a discontinuous pattern of background classification is associated with poor outcome [3, 17, 18]. However this pattern can be difficult to detect especially in preterm infants and can be misdiagnosed due to artefactual findings [14, 19, 20]. By contrast, the sleep-wake cycle can be used practically for monitoring brain function in both term and preterm infants > 28 weeks of gestation.

The presence of cyclicity in aEEG can predict an infant’s neurologic outcome [3, 18]. The appearance of cyclicity has high prognostic value of better neurologic outcome with 74.5 % to 96.1 % sensitivity and 100 % specificity [5, 14, 21]; while the absence of sleep-wake pattern had 46.8 % sensitivity and 100 % specificity for predicting poor neurologic outcome [5]. Cyclicity within 72 h after birth is especillay predictive of a good outcome [15, 21, 22]. Term infants with cyclicity within 36 h after birth have good neurodevelopmental outcomes [14]. One study reported that the emergence of sleep stages has no prognostic value for 2-year neurologic outcome [4]. Preterm infants with severe cerebral lesions exhibit maturation delays with regard to aEEG cyclic activity [7, 14, 15, 23]. Moreover, poor presentation of cyclicity in preterm infants is associated with brain lesions [24].

Our study supports these conclusions through the result of earlier presence of sleep-wake cycling in the favorable neuroimaging group. Specifically, this study focuses on the latency of sleep-wake cycling to neuroimaging outcome, as compared to the most previous studies that are focused on the relationship between presence [14, 21] or maturation [15, 24, 25] of sleep-wake cycling and prognosis. Our present study has additional advantages. First, aEEG recording were repeated sufficiently over long periods of time; every 1 or 2 weeks until the cyclicity developed. As a result, we could reasonably determine that sleep-wake cycles appeared later in most patients in the severe brain imaging group. Moreover, we could suggest an optimal aEEG follow-up time to predict unfavorable brain image which can be related with worse neurologic outcomes. If there was no cyclicity on aEEG within the first 24 h, follow-up recording at 12 days after birth is recommended.

Our results indicate that the sleep-wake cycling begins later than had previously been reported in previous studies [15, 16]. The difference seems to be a result of the study populations following various definitions for patient groups for perinatal asphyxia or hypoxic ischemic encephalopathy in the other studies. The first few hours after birth are stressful, especially for preterm infants who can experience respiratory distress syndrome, cold stress, mechanical ventilation, invasive procedures, pain, or new environments that prevent the establishment of a normal sleep pattern. Moreover, Hoppenbrouwers et al. found that babies on ventilators had different sleep patterns [26]. Other possible causes of late detection of sleep-wake cycling are skipping a crucial time period of monitoring and stringent classification of cyclicity.

One of the limitations of this study was that we only examined serial neuroimages after birth without long-term neurodevelopmental follow-up. In addition, brain MRI was not performed in the most patients. It is possible that a crucial time period of their sleep-wake cycle development was unobserved because aEEG was not continuously recorded. Raw EEG was not analyzed in every case although several studies report that some pattern in conventional EEG can assess the patient’s sleep and brain status [27, 28]. Lastly, the number of preterm infants <32 weeks of gestation were not sufficient; further, most of these patients were enrolled into Group II. Nevertheless, we found favorable brain images to be more powerfully associated with the early presentation of cyclicity as compared to the mature gestational age. For the next step, a larger scale longer-term follow-up study to reveal the neurodevelopmental outcomes of preterm infants is required to verify our findings.

## Conclusion

Our study demonstrated that an earlier presentation of cyclicity on aEEG strongly correlates with favorable neuroimages for the newborn infants who had the possibility of perinatal asphyxia. Moreover, absence of definite cyclicity on aEEG from the day of birth through 12 days after birth is considered as the potential risk factor of adverse neurologic outcome.

## Abbreviations

aEEG: Amplitude-integrated electroencephalography
CI: Confidence interval
EEG: Electroencephalography
GA: Gestational age
GMH: Germinal matrix hemorrhage
HIE: Hypoxic ischemic encephalopathy
IVH: Intraventricular hemorrhage
PVE: Periventricular echodensity
PVL: Periventricular leukomalacia
